# Supplementary material for: Association of cystatin C with 20-year mortality risk in the general US population: a cohort study
Source: Ren Fail. 2025 Sep 22;47(1):2549401. doi: 10.1080/0886022X.2025.2549401 (PMC12456039; doi:10.1080/0886022X.2025.2549401)
Supplement: Clean copy of LRNF2025RL0028 R1.docx [file IRNF_A_2549401_SM6605.docx]

**Association of Cystatin C with 20-Year Mortality Risk in the General US Population: A Cohort Study**

Yujuan Yuan MD^1,2#^, Zitong Guo PHD^1,2#^, Xiangyu Dong MS^1,2^, Jie Gao MS^1,2^, Zhao Wang MD^1,2^, Yining Yang PHD^1,2*^

1. Department of Cardiology, People’s Hospital of Xinjiang Uyghur Autonomous Region, Urumqi, Xinjiang, China.
2. Xinjiang Key Laboratory of Cardiovascular Homeostasis and Regeneration Research, Urumqi,Xinjiang ,China.

# The authors contributed equally to this study.

* Correspondence: Yining Yang, yangyn5126@163.com. No.91 Tianchi Road, Urumqi 830001, Xinjiang, China.

**Abstract**

**Background:** Impaired kidney function is associated with increased mortality. This study evaluated the association between serum cystatin C levels and long-term all-cause, cardiovascular, and cerebrovascular mortality in the general U.S. population.

**Methods:** Impaired kidney function is associated with increased mortality. This study evaluated the association between serum cystatin C levels and long-term all-cause, cardiovascular, and cerebrovascular mortality in the general U.S. population.

**Results:** During a median follow-up of 207 months (interquartile range [IQR]: 188.0–227.0), 3,538 participants died. The median baseline age was 44.0 years (IQR: 33.0–57.0). Participants were categorized into quintiles based on serum cystatin C, creatinine, and estimated glomerular filtration rate (eGFR). Multivariate analysis, revealed a significant association of cystatin C’s third, fourth, and fifth quintiles with an increased risk of all-cause mortality. Compared to the first quintile, the hazard ratios (HR) [95% confidence intervals (CI)] for all-cause mortality were as follows: second quintile, 1.05 (0.82-1.34); third quintile, 1.29 (1.03-1.60); fourth quintile, 1.46 (1.17-1.83); and fifth quintile, 2.32 (1.86-2.89). Furthermore, the second quintile had a 1.78-fold risk (HR: 1.78, 95%CI: 1.11-2.86), the fourth quintile had a 1.85-fold risk (HR: 1.85, 95% CI: 1.17- 2.92), and the fifth quintile had a 2.97-fold risk of cardiovascular mortality (HR: 2.97, 95% CI: 1.89-4.68). However, adjusted analysis did not reveal creatinine and eGFR as predictors for any outcome. After adjusting for confounding factors, creatinine and eGFR were not significantly associated with the risk of death.

**Conclusion:** Cystatin C emerged as an independent predictor of all-cause and cardiovascular mortality with in the general population of the US.

**Keywords: Chronic kidney disease, Cystatin C, All-cause mortality, Cardiovascular mortality, General population.**

**Introduction**

Impaired kidney function and chronic kidney disease (CKD) have been associated with heightened risks of all-cause and cardiovascular mortality [1-5]. The Global Burden of Disease Study indicates the substantial impact of impaired kidney function, with 1.4 million deaths attributed to cardiovascular disease and 1.2 million deaths attributed to CKD in 2017, constituting 4.6% of total mortality [6].

Serum creatinine levels, commonly used as a biomarker for estimating glomerular filtration rate (eGFR), exhibit delayed responses to renal damage [7]. It can be influenced by various factors such as age, gender, race, physical activity, and protein intake [8-11]. However, creatinine-based equations for eGFR tend to overestimate GFR and underestimate the presence or severity of CKD in older individuals. They are insensitive markers of kidney impairment in early disease stages [12-14].

Cystatin C, a 13-kDa cysteine protease inhibitor consisting of 122 amino acids, has emerged as a more precise indicator of GFR than creatinine. Its serum concentrations are independent of muscle mass and are not influenced by age or gender [15, 16]. While cystatin C has demonstrated predictive value for cardiovascular and non-cardiovascular mortality in specific populations such as patients with diabetes [17], stroke [18, 19], CKD patients [20] and the older population [14, 21], its predictive capacity for long-term mortality outcomes in the general population has not been extensively investigated. This study examines the association between cystatin C levels and long-term mortality associated with all-cause, cardiovascular and cerebrovascular diseases among the general population of the US. Moreover, the associations of cystatin C, creatinine, and eGFR with the risk of all outcomes within this cohort were examined.

**Materials and Methods**

**Participants and study design**

This is a retrospective cohort study utilizing prospectively collected data from the National Health and Nutrition Examination Survey (NHANES) 1999–2004, in which Cystatin C results were available (https://wwwn.cdc.gov/nchs/nhanes/). The NHANES was assembled to evaluate the health and nutritional status among adults and children in the US, with data collected through home interviews and standardized physical mobile examinations conducted at mobile examination centers, released in 2-year cycles. There were 31,126 participants from three cycles between 1999-2000 and 2003-2004. Participants aged<20 years at enrolment, for whom serum samples for cystatin C and creatinine measurement were unavailable (n=2,979). Further exclusion was performed on participants lacking time-to-event data for mortality due to insufficient identifying information during mortality data linkage (n=13). The final analytical sample comprised 12,340 participants **[Fig.1]**.

**Measurement of variables**

Serum cystatin C levels were measured using a Cystatin C immunoassay (Siemens Healthcare Diagnostics, Erlangen, Germany) on an automated multi-channel analyzer, Siemens Dimension Vista 1500 (Siemens Healthcare Diagnostics, Erlangen, Germany), with lower and upper detection limits of 0.23 mg/L and 8.00 mg/L, respectively. Creatinine concentration in serum was measured using the Jaffe rate method (kinetic alkaline picrate) on the LX20 modular chemistry side. Age groups were categorized into 20 to 44, 45 to 59, and ≥60 years. Race was classified as Mexican American, other Hispanic, non-Hispanic White, non-Hispanic Black, and other races. Marital status was categorized as widowed, divorced, separated, never married, and living with a partner. Education attainment was classified as less than ninth grade, 9–11th grade, high school graduate/ General Educational Development (GED) or equivalent, some college or Associate of Arts (AA) degree, and college graduate or above. Body Mass Index (BMI) categories were defined as normal < 25.0, overweight 25.0–29.9, and obese ≥30kg/m^2^. Smoking status was categorized as never, current, and former smoker. Drinking status was classified as never, light, moderate, and heavy alcohol consumption, respectively, based on daily drinking volume (0, <1, 1–8, and >8 drinks per week). GFR was calculated separately using the CKD-EPI creatinine and cystatin C equations, which have demonstrated superior accuracy in mortality risk stratification compared to the traditional Modification of Diet in Renal Disease formula [22].

**Outcomes**

The primary outcome assessed was all-cause mortality, with cardiovascular and cerebrovascular mortality as secondary outcomes. All-cause mortality data were obtained by linking to the National Death Index until December 31, 2019, with mortality data linked using assigned sequence numbers. Follow-up time was defined as the duration in person-months from the interview data to the date of mortality or the end of the follow-up period. Cardiovascular mortality was defined as death due to heart disease, based on UCOD_113 codes 54–68 under the ICD-10 system, encompassing ischemic heart disease, heart failure, and other related conditions. Cerebrovascular mortality was defined as death due to cerebrovascular diseases, corresponding to UCOD_113 code 70, including both ischemic and hemorrhagic stroke. These definitions followed the classification system adopted by the National Center for Health Statistics in the NHANES Linked Mortality Files.

**Statistical analysis**

NHANES studies utilize complex survey designs, rendering standard estimates inappropriate. Therefore, all analyses were meticulously weighted to ensure the representativeness of the US population. Continuous variables are presented as weighted medians [interquartile range (IQR)], while categorical variables are expressed as weighted percentages. Baseline characteristics were analyzed using with the Wilcoxon rank-sum test for continuous variables and the chi-square test for categorical variables. The study population was stratified into quintiles based on cystatin C, creatinine levels, and eGFR to examine the association between renal measurements and outcomes. Cox proportional hazard models were employed to evaluate the association of each renal measure with the outcomes. Covariates, including age, sex, BMI, race, smoking and drinking status, history of hypertension, diabetes, coronary artery disease, stroke, and cancer, were consistently included in the models for all outcomes. Weighted Kaplan–Meier curves assessed the differences across cystatin C quintiles. At the same time, weighted restrictive cube splines were employed to model the association between cystatin C and each outcome flexibly in adjusted analyses. Subgroup analyses were conducted according to sex, age group (18-59, vs ≥60 years), eGFR (<60 vs ≥60 mL/min/1.73 m² calculated using the CKD-EPI 2012 cystatin C equation), smoking status (yes vs no), history of CAD (yes vs no), and stroke (yes vs no) . To assess the robustness of our primary findings, we conducted a of sensitivity analyse by excluding participants with follow-up durations of less than 2 years. All statistical analyses were performed using R software. A two-sided *P* value < 0.05 was considered to indicate statistical significance.

**Results**

**Baseline Characteristics According to Cystatin C**

Among the 12,340 participants in the study, 6,423 were men and 5,917 were women. The median baseline age was 44.0 years (IQR: 33.0–57.0). Participants were categorized into five groups based on quintiles of cystatin C: quintile 1 (<0.640 mg/L), quintile 2 (0.640–0.717 mg/L), quintile 3 (0.718–0.799 mg/L), quintile 4 (0.800–0.932 mg/L), and quintile 5 (>0.932 mg/L). Compared to participants in Quintile 1 (lowest cystatin C), those in higher quintiles had a lower proportion of females (from 71.5% to 51.7%), a higher proportion of current smokers (17.3% to 24.2%), a higher proportion of non-Hispanic Whites (54.9% to 81.8%), and a lower proportion of moderate/heavy drinkers (36.5% to 25.3%) **[Table 1]**.

**Association Between Each Renal Measurement and All-cause Mortality**

Analysis of mortality incidence for all-cause mortality across quintiles of cystatin C, creatinine, and eGFR revealed substantial differences [**Fig. 2**]. Cystatin C and eGFR categories exhibited a significant and linear association with the risk of all-cause mortality, whereas serum creatinine did not. Over the follow up period, with a median time of 207 months (IQR: 188.0, 227.0); 3538 partcipants experienced mortality. The Kaplan–Meier curve indicated that participants in the second to fifth quintiles of cystatin C had a higher risk of all-cause mortality compared to those in the first quintile (log-rank test, P<0.001) [**Fig. 3**].

Multivariate analysis revealed that cystatin C’s third, fourth, and fifth quintiles were significantly associated with the risk of all-cause mortality, while the second quintile showed no significance [**Table 2**]. Compared to the first quintile, the hazard ratios (HR) [95% confidence intervals (CI)] for all-cause mortality were as follows: second quintile, 1.05 (0.82 to 1.34); third quintile, 1.29 (1.03 to 1.60); fourth quintile, 1.46 (1.17 to 1.83); and fifth quintile, 2.32 (1.86 to 2.89). Regarding creatinine and eGFR, the second to fifth quintiles did not increase the risk of all-cause mortality compared to the lowest quintile in the adjusted model [**Table supplement 1**].

**Association Between Each Renal Measurement and Cardiovascular and Cerebrovascular Mortality**

During the follow-up, 1,140 participants experienced cardiovascular mortality and 202 participants experienced cerebrovascular mortality. In adjusted analysis, compared to the first quintile of cystatin C, the second quintile had a 1.78-fold risk (HR: 1.78, 95% CI: 1.11 to 2.86), the fourth quintile had a 1.85-fold risk (HR: 1.85, 95% CI: 1.17 to 2.92), and the fifth quintile had a 2.97-fold risk of cardiovascular mortality (HR: 2.97, 95% CI: 1.89 to 4.68) [**Table 2**]. Unadjusted analysis showed a significant increase in the risk of cardiovascular mortality for creatinine and eGFR. However, in adjusted analysis, creatinine and eGFR did not significantly correlate with cardiovascular mortality [**Table supplement 1**].

In an unadjusted analysis of cerebrovascular mortality, the fourth and fifth quintile of cystatin C exhibited increased risk compared to the lowest quintile. However, no significant associations persisted after multivariate adjustment. Moreover, creatinine and eGFR showed no significant association with cerebrovascular mortality in the adjusted analyses [**Table supplement 1**].

**Association Between Cystatin C and All-cause Mortality, Cardiovascular and Cerebrovascular Mortality**

Restrictive cubic spline analysis revealed a non-linear relationship between cystatin C and all-cause mortality, cardiovascular, and cerebrovascular mortalities after adjusting for potential confounding factors [**Fig. 4**]. Furthermore, subgroups were categorized into low-risk (quintiles 1 and 2), intermediate-risk (quintiles 3 and 4), and high-risk (quintile 5) based on the results from Table 2, corresponding to cystatin C levels of <0.718 mg/L, 0.718 to 0.932 mg/L, and >0.932 mg/L. Compared with the low-risk group (cystatin C <0.718 mg/L), the intermediate-risk and high-risk groups significantly increased the risk of all-cause mortality. Moreover, the high-risk group exhibited a higher risk of cardiovascular mortality than the low-risk group. However, the intermediate-risk group did not show a significantly higher risk than the low-risk group. For cerebrovascular mortality, being in the intermediate-risk or high-risk group was not independently associated with a greater risk **[Table 3]**. Notably, the results remained consistent when estimated glomerular filtration rate (eGFR) was calculated using the CKD-EPI 2012 cystatin C equation [**Table supplement 2**].

**Subgroups and Sensitivity Analysis**

Elevated serum cystatin C levels were associated with increased all-cause mortality across most subgroups. Compared with levels <0.718 mg/L, cystatin C levels of 0.718–0.932 mg/L and >0.932 mg/L were linked to higher risks, particularly among individuals aged >59 years (HRs: 1.545 and 3.458), current smokers (1.484 and 2.201), and those with hypertension (1.401 and 2.279). These associations were consistent across sexes and among individuals with preserved kidney function (eGFR ≥60 mL/min/1.73 m²), but not significant in those with eGFR <60 mL/min/1.73 m² [**Figure supplement 1**].

Sensitivity analyses confirmed the robustness of the findings. After excluding participants with follow-up <2 years, the dose–response relationships between cystatin C and all-cause, cardiovascular, and cerebrovascular mortality remained stable [**Table supplement 3**].

**Discussion**

This study demonstrates that serum cystatin C is a strong and independent predictor of long-term all-cause and cardiovascular mortality in the US general population. In contrast, serum creatinine and eGFR showed no independent association with these outcomes after adjustment. Our findings suggest that cystatin C provides a more reliable measure of mortality risk than traditional renal function markers. To our knowledge, this is one of the few large, nationally representative cohort studies to directly compare these renal biomarkers over a 17-year follow-up period, offering novel insights into population-level risk stratification.

Our results align with previous studies demonstrating that cystatin C could predict all-cause and cardiovascular mortalities. However, many of these studies were conducted on specific populations. For instance, Shlipak et al. demonstrated that cystatin C levels are associated with an increased risk of mortality and cardiovascular events in older individuals [3]. Another study identified serum cystatin C as an independent predictor of long-term mortality in populations with relatively normal renal function [23]. Furthermore, a meta-analysis revealed that serum cystatin C is independently associated with adverse vascular outcomes in participants with suspected or established coronary artery disease in terms of all-cause and cardiovascular mortalities, independent of creatinine-based eGFR [24]. Although cystatin C has shown greater long-term prognostic value than eGFR or creatinine in some cohorts and studies, its clinical significance is not yet widely recognized in the medical community. Our study extends these findings by demonstrating that serum creatinine levels and eGFR were not associated with the risk of all outcomes examined after multivariate adjustment. In contrast, cystatin C levels were strongly associated with the risk of all-cause and cardiovascular mortalities. The results of this 20-year cohort follow-up study will help to validate and enhance the appreciation of cystatin C's prognostic importance in the general population. This association suggests that numerous factors influence serum creatinine and creatinine-based eGFR.

Consistent with previous analyses in older persons without CKD, cystatin C is a superior prognostic biomarker for the risk of death, cardiovascular disease, and CKD compared to serum creatinine and eGFR [14, 25]. Cystatin C emerged as a more accurate estimate of eGFR than serum creatinine across different populations [7, 26-28]. Existing evidences suggests that cystatin C-based estimation of CKD offers superior prognostic value and more accurate prediction of cardiovascular diseases, cardiovascular death, and all-cause mortality than creatinine-based estimation [7, 12, 25, 29]. Cystatin C-based eGFR demonstrates greater sensitivity and specificity for assessing cardiovascular disease and mortality risks, particularly in mild CKD [30].

Several potential mechanisms may underlie the observed association between elevated cystatin C levels and long-term mortality. Elevated cystatin C has been linked to an increased risk of developing chronic kidney disease (CKD), which is itself a well-established risk factor for all-cause and cardiovascular mortality. Therefore, cystatin C may serve as an early marker of kidney dysfunction that contributes to adverse outcomes. In addition, prior studies have suggested that cystatin C may reflect "pre-clinical" renal impairment not captured by creatinine-based eGFR alone [14, 23]. Moreover, cystatin C is a known inhibitor of cysteine proteases such as cathepsins, which have been implicated in the development of atherosclerosis[31]. Elevated levels of circulating cathepsin S have been associated with increased risks of cardiovascular events and mortality in previous research [32, 33]. Although these pathways offer plausible biological explanations, our study did not directly assess markers of atherosclerosis or protease activity. Further investigation is warranted to elucidate the specific mechanistic pathways linking cystatin C to mortality, particularly among individuals with preserved creatinine-based eGFR.

Our study has certain limitations. Firstly, despite conducting multivariate analyses, there may still be unmeasurable confounding factors inherent to the observational study design. Secondly, the NHANES data only provided baseline levels of cystatin C and covariates, lacking ambulatory monitoring, thereby precluding the consideration of changes in exposure and covariate status during follow-up. Thirdly, an observational study cannot effectively establish causal relationships between cystatin C and mortality. Future investigations could employ Mendelian randomisation and other research methodologies to corroborate the observed association. Ultimately, since the results predominantly relate to the US population, their generalizability to other populations might be restricted, although they provide some reference value.

**Conclusions:** This study demonstrated a positive association of serum cystatin C levels with all-cause and cardiovascular mortalities with in the US population. The 20-year longitudinal cohort study underscores the significance of cystatin C as a predictive biomarker for mortality in the general population.

**Data availability statement**

The data presented in the study are taken from the NHANES website, linked to [https://www.cdc.gov/nchs/nhanes/index.htm](https://www.cdc.gov/nchs/nhanes/index.htm).

**Ethics approval and consent to participate**

The NHANES protocols were approved by the National Center for Health Statistics Ethics. Review Board of the US CDC, and written informed consent from all the participants was provided during the survey.

**Consent for publication**

Not applicable

**Availability of data and materials**

The data that support the findings of this study are available from the corresponding author on reasonable request.

**Competing interests**

The authors declare that they have no known competing financial interests or personal relationships that could have appeared to influence the work reported in this paper.

**Funding**

This work was supported by Natural Science Foundation of Xinjiang Uygur Autonomous Region (2022D01C634) and Tianshan Talent Cultivation Program Project of Xinjiang Uygur Autonomous Region (2022TSYCLJ0028).

**Authors' contributions**

Yujuan Yuan and Zitong Guo had participated in the whole study, in terms of designing the work, collecting and analysing data, writing the first manuscript. Professor Yang conceived the design and revised the manuscript. Xiangyu Dong, Jie Gao, Zhao Wang, gave advice on the study analysis.

References

1. Go AS, Chertow GM, Fan D, McCulloch CE, Hsu CY: **Chronic kidney disease and the risks of death, cardiovascular events, and hospitalization**. *N Engl J Med* 2004, **351**(13):1296-1305.

2. Rifkin DE, Katz R, Chonchol M, Fried LF, Cao J, de Boer IH, Siscovick DS, Shlipak MG, Sarnak MJ: **Albuminuria, impaired kidney function and cardiovascular outcomes or mortality in the elderly**. *Nephrol Dial Transplant* 2010, **25**(5):1560-1567.

3. Shlipak MG, Sarnak MJ, Katz R, Fried LF, Seliger SL, Newman AB, Siscovick DS, Stehman-Breen C: **Cystatin C and the risk of death and cardiovascular events among elderly persons**. *N Engl J Med* 2005, **352**(20):2049-2060.

4. Matsushita K, Ballew SH, Wang AY, Kalyesubula R, Schaeffner E, Agarwal R: **Epidemiology and risk of cardiovascular disease in populations with chronic kidney disease**. *Nat Rev Nephrol* 2022, **18**(11):696-707.

5. Zanoli L, Lentini P, Briet M, Castellino P, House AA, London GM, Malatino L, McCullough PA, Mikhailidis DP, Boutouyrie P: **Arterial Stiffness in the Heart Disease of CKD**. *J Am Soc Nephrol* 2019, **30**(6):918-928.

6. Collaboration GBDCKD: **Global, regional, and national burden of chronic kidney disease, 1990-2017: a systematic analysis for the Global Burden of Disease Study 2017**. *Lancet* 2020, **395**(10225):709-733.

7. Zou LX, Sun L, Nicholas SB, Lu Y, K SS, Hua R: **Comparison of bias and accuracy using cystatin C and creatinine in CKD-EPI equations for GFR estimation**. *Eur J Intern Med* 2020, **80**:29-34.

8. Yoo JJ, Kim SG, Kim YS, Lee B, Lee MH, Jeong SW, Jang JY, Lee SH, Kim HS, Kim YD *et al*: **Estimation of renal function in patients with liver cirrhosis: Impact of muscle mass and sex**. *J Hepatol* 2019, **70**(5):847-854.

9. Hansen SI, Petersen PH, Lund F, Fraser CG: **Plasma creatinine medians from patients partitioned by gender and age used as a tool for assessment of analytical stability at different concentrations**. *Clin Chem Lab Med* 2019, **58**(1):69-76.

10. Inker LA, Eneanya ND, Coresh J, Tighiouart H, Wang D, Sang Y, Crews DC, Doria A, Estrella MM, Froissart M *et al*: **New Creatinine- and Cystatin C-Based Equations to Estimate GFR without Race**. *N Engl J Med* 2021, **385**(19):1737-1749.

11. Levey AS, Titan SM, Powe NR, Coresh J, Inker LA: **Kidney Disease, Race, and GFR Estimation**. *Clin J Am Soc Nephrol* 2020, **15**(8):1203-1212.

12. Lees JS, Welsh CE, Celis-Morales CA, Mackay D, Lewsey J, Gray SR, Lyall DM, Cleland JG, Gill JMR, Jhund PS *et al*: **Glomerular filtration rate by differing measures, albuminuria and prediction of cardiovascular disease, mortality and end-stage kidney disease**. *Nat Med* 2019, **25**(11):1753-1760.

13. Stevens LA, Coresh J, Greene T, Levey AS: **Assessing kidney function--measured and estimated glomerular filtration rate**. *N Engl J Med* 2006, **354**(23):2473-2483.

14. Shlipak MG, Katz R, Sarnak MJ, Fried LF, Newman AB, Stehman-Breen C, Seliger SL, Kestenbaum B, Psaty B, Tracy RP *et al*: **Cystatin C and prognosis for cardiovascular and kidney outcomes in elderly persons without chronic kidney disease**. *Ann Intern Med* 2006, **145**(4):237-246.

15. Benoit SW, Ciccia EA, Devarajan P: **Cystatin C as a biomarker of chronic kidney disease: latest developments**. *Expert Rev Mol Diagn* 2020, **20**(10):1019-1026.

16. Pottel H, Bjork J, Rule AD, Ebert N, Eriksen BO, Dubourg L, Vidal-Petiot E, Grubb A, Hansson M, Lamb EJ *et al*: **Cystatin C-Based Equation to Estimate GFR without the Inclusion of Race and Sex**. *N Engl J Med* 2023, **388**(4):333-343.

17. Tsai CW, Grams ME, Inker LA, Coresh J, Selvin E: **Cystatin C- and creatinine-based estimated glomerular filtration rate, vascular disease, and mortality in persons with diabetes in the U.S**. *Diabetes Care* 2014, **37**(4):1002-1008.

18. Hojs Fabjan T, Penko M, Hojs R: **Newer glomerular filtration rate estimating equations for the full age spectrum based on serum creatinine and cystatin C in predicting mortality in patients with ischemic stroke**. *Eur J Intern Med* 2018, **52**:67-72.

19. Zehetner A: **Bilateral natal clefts in a neonate**. *J Paediatr Child Health* 2013, **49**(9):786, 791.

20. Peralta CA, Katz R, Sarnak MJ, Ix J, Fried LF, De Boer I, Palmas W, Siscovick D, Levey AS, Shlipak MG: **Cystatin C identifies chronic kidney disease patients at higher risk for complications**. *J Am Soc Nephrol* 2011, **22**(1):147-155.

21. Salminen M, Laine K, Korhonen P, Wasen E, Vahlberg T, Isoaho R, Kivela SL, Lopponen M, Irjala K: **Biomarkers of kidney function and prediction of death from cardiovascular and other causes in the elderly: A 9-year follow-up study**. *Eur J Intern Med* 2016, **33**:98-101.

22. Matsushita K, Mahmoodi BK, Woodward M, Emberson JR, Jafar TH, Jee SH, Polkinghorne KR, Shankar A, Smith DH, Tonelli M *et al*: **Comparison of risk prediction using the CKD-EPI equation and the MDRD study equation for estimated glomerular filtration rate**. *JAMA* 2012, **307**(18):1941-1951.

23. Wu CK, Lin JW, Caffrey JL, Chang MH, Hwang JJ, Lin YS: **Cystatin C and long-term mortality among subjects with normal creatinine-based estimated glomerular filtration rates: NHANES III (Third National Health and Nutrition Examination Survey)**. *J Am Coll Cardiol* 2010, **56**(23):1930-1936.

24. Yang S, Song L, Zhao L, Dong P, Lai L, Wang H: **Predictive value of cystatin C in people with suspected or established coronary artery disease: A meta-analysis**. *Atherosclerosis* 2017, **263**:60-67.

25. Shlipak MG, Matsushita K, Arnlov J, Inker LA, Katz R, Polkinghorne KR, Rothenbacher D, Sarnak MJ, Astor BC, Coresh J *et al*: **Cystatin C versus creatinine in determining risk based on kidney function**. *N Engl J Med* 2013, **369**(10):932-943.

26. Helmersson-Karlqvist J, Lipcsey M, Arnlov J, Bell M, Ravn B, Dardashti A, Larsson A: **Addition of cystatin C predicts cardiovascular death better than creatinine in intensive care**. *Heart* 2022, **108**(4):279-284.

27. Pinsino A, Mondellini GM, Royzman EA, Hoffman KL, D'Angelo D, Mabasa M, Gaudig A, Zuver AM, Masoumi A, Garan AR *et al*: **Cystatin C- Versus Creatinine-Based Assessment of Renal Function and Prediction of Early Outcomes Among Patients With a Left Ventricular Assist Device**. *Circ Heart Fail* 2020, **13**(1):e006326.

28. Li H, Wang A, Qi G, Guo J, Li X, Wang W, Xiang Y, Guo X: **Cystatin C and risk of new-onset depressive symptoms among individuals with a normal creatinine-based estimated glomerular filtration rate: A prospective cohort study**. *Psychiatry Res* 2019, **273**:75-81.

29. Rothenbacher D, Rehm M, Iacoviello L, Costanzo S, Tunstall-Pedoe H, Belch JJF, Soderberg S, Hultdin J, Salomaa V, Jousilahti P *et al*: **Contribution of cystatin C- and creatinine-based definitions of chronic kidney disease to cardiovascular risk assessment in 20 population-based and 3 disease cohorts: the BiomarCaRE project**. *BMC Med* 2020, **18**(1):300.

30. Lees JS, Rutherford E, Stevens KI, Chen DC, Scherzer R, Estrella MM, Sullivan MK, Ebert N, Mark PB, Shlipak MG: **Assessment of Cystatin C Level for Risk Stratification in Adults With Chronic Kidney Disease**. *JAMA Netw Open* 2022, **5**(10):e2238300.

31. Sukhova GK, Shi GP, Simon DI, Chapman HA, Libby P: **Expression of the elastolytic cathepsins S and K in human atheroma and regulation of their production in smooth muscle cells**. *J Clin Invest* 1998, **102**(3):576-583.

32. Jobs E, Ingelsson E, Riserus U, Nerpin E, Jobs M, Sundstrom J, Basu S, Larsson A, Lind L, Arnlov J: **Association between serum cathepsin S and mortality in older adults**. *JAMA* 2011, **306**(10):1113-1121.

33. Wuopio J, Hilden J, Bring C, Kastrup J, Sajadieh A, Jensen GB, Kjoller E, Kolmos HJ, Larsson A, Jakobsen JC *et al*: **Cathepsin B and S as markers for cardiovascular risk and all-cause mortality in patients with stable coronary heart disease during 10 years: a CLARICOR trial sub-study**. *Atherosclerosis* 2018, **278**:97-102.

**Table 1 Weighted baseline characteristics according to Quintiles of Cysatin C group**

| **Characteristic** | **Overall**  **N=12340** | **Quintile 1**  **(<0.640 mg/L)**  **N=2485** | **Quintile 2**  **(0.640–0.717 mg/L)**  **N = 2460** | **Quintile 3 (0.718–0.799 mg/L)**  **N = 2481** | **Quintile 4 (0.800–0.932 mg/L)**  **N = 2458** | **Quintile 5**  **(>0.932 mg/L)**  **N = 2456** |
| --- | --- | --- | --- | --- | --- | --- |
| **Age (years)** | 44.0 (33.0, 57.0) | 36.0 (28.0, 44.0) | 39.0 (30.0, 49.0) | 43.0 (32.0, 54.0) | 50.0 (37.0, 63.0) | 68.0 (54.0, 77.0) |
| **Age group (n, %)** |  |  |  |  |  |  |
| *<45* | 5,456.0 (50.7%) | 1,870.0 (75.2%) | 1,502.0 (63.8%) | 1,147.0 (53.1%) | 727.0 (38.6%) | 210.0 (13.1%) |
| *45-59* | 2,522.0 (26.7%) | 432.0 (20.9%) | 596.0 (28.4%) | 632.0 (30.4%) | 580.0 (31.0%) | 282.0 (20.5%) |
| *＞59* | 4,362.0 (22.5%) | 183.0 (3.9%) | 362.0 (7.8%) | 702.0 (16.5%) | 1,151.0 (30.4%) | 1,964.0 (66.5%) |
| **Sex** |  |  |  |  |  |  |
| Female | 6,423.0 (52.2%) | 1,777.0 (71.5%) | 1,293.0 (54.7%) | 1,106.0 (43.5%) | 1,057.0 (41.3%) | 1,190.0 (51.7%) |
| Male | 5,917.0 (47.8%) | 708.0 (28.5%) | 1,167.0 (45.3%) | 1,375.0 (56.5%) | 1,401.0 (58.7%) | 1,266.0 (48.3%) |
| **Race (n, %)** |  |  |  |  |  |  |
| Mexican American | 2,783.0 (7.3%) | 787.0 (13.6%) | 637.0 (9.2%) | 549.0 (6.2%) | 440.0 (4.0%) | 370.0 (2.5%) |
| Other Hispanic | 567.0 (5.7%) | 132.0 (7.1%) | 122.0 (6.0%) | 111.0 (5.5%) | 125.0 (5.7%) | 77.0 (3.9%) |
| Non-Hispanic White | 6,361.0 (71.6%) | 848.0 (54.9%) | 1,113.0 (68.3%) | 1,301.0 (74.5%) | 1,492.0 (80.1%) | 1,607.0 (81.8%) |
| Non-Hispanic Black | 2,195.0 (10.9%) | 600.0 (17.6%) | 490.0 (11.6%) | 437.0 (9.7%) | 333.0 (7.1%) | 335.0 (8.3%) |
| Other Race | 434.0 (4.5%) | 118.0 (6.8%) | 98.0 (4.9%) | 83.0 (4.1%) | 68.0 (3.1%) | 67.0 (3.5%) |
| **Married status (n, %)** |  |  |  |  |  |  |
| Married | 7,001.0 (58.1%) | 1,403.0 (55.6%) | 1,368.0 (57.5%) | 1,447.0 (59.7%) | 1,480.0 (62.0%) | 1,303.0 (54.4%) |
| Widowed | 1,236.0 (6.6%) | 55.0 (2.1%) | 83.0 (2.3%) | 153.0 (3.5%) | 283.0 (7.4%) | 662.0 (22.4%) |
| Divorced | 1,091.0 (9.5%) | 169.0 (7.5%) | 223.0 (9.9%) | 225.0 (9.7%) | 227.0 (9.2%) | 247.0 (11.5%) |
| Separated | 385.0 (2.6%) | 98.0 (3.5%) | 87.0 (2.7%) | 85.0 (2.7%) | 65.0 (2.3%) | 50.0 (1.8%) |
| Never married | 1,921.0 (17.2%) | 559.0 (23.3%) | 527.0 (21.0%) | 413.0 (17.9%) | 278.0 (13.6%) | 144.0 (7.3%) |
| Living with partner | 706.0 (6.0%) | 201.0 (8.1%) | 172.0 (6.5%) | 158.0 (6.5%) | 125.0 (5.6%) | 50.0 (2.6%) |
| **Education (n, %)** |  |  |  |  |  |  |
| Less than 9th grade | 1,925.0 (7.0%) | 324.0 (6.4%) | 303.0 (5.5%) | 326.0 (5.0%) | 398.0 (6.9%) | 574.0 (13.4%) |
| 9-11th grade | 2,059.0 (13.5%) | 435.0 (12.9%) | 399.0 (12.3%) | 385.0 (12.1%) | 411.0 (13.9%) | 429.0 (17.7%) |
| High school graduate/GED or equivalent | 2,944.0 (26.2%) | 514.0 (20.9%) | 585.0 (25.4%) | 617.0 (27.2%) | 614.0 (28.4%) | 614.0 (29.4%) |
| Some college or AA degree | 3,128.0 (29.4%) | 644.0 (29.4%) | 696.0 (31.0%) | 671.0 (31.6%) | 635.0 (30.5%) | 482.0 (22.3%) |
| College graduate or above | 2,284.0 (23.9%) | 568.0 (30.4%) | 477.0 (25.8%) | 482.0 (24.0%) | 400.0 (20.3%) | 357.0 (17.2%) |
| **BMI (kg/㎡)** | 27.18 (23.80, 31.31) | 25.26 (22.28, 28.94) | 26.79 (23.51, 30.40) | 27.68 (24.22, 31.84) | 28.14 (24.73, 32.52) | 28.33 (24.78, 32.99) |
| **BMI group (n, %)** |  |  |  |  |  |  |
| Normal or low weight<25 | 3,887.0 (34.0%) | 1,036.0 (48.0%) | 829.0 (36.7%) | 709.0 (31.0%) | 638.0 (27.2%) | 675.0 (26.0%) |
| Overweight 25-29.99 | 4,456.0 (34.7%) | 841.0 (31.8%) | 929.0 (36.7%) | 894.0 (34.9%) | 921.0 (35.3%) | 871.0 (34.2%) |
| Obese ≥30 | 3,997.0 (31.3%) | 608.0 (20.2%) | 702.0 (26.7%) | 878.0 (34.1%) | 899.0 (37.5%) | 910.0 (39.8%) |
| **Weight (kg)** | 77.8 (65.9, 91.5) | 69.4 (59.6, 81.6) | 77.0 (65.8, 89.2) | 80.7 (69.2, 95.4) | 81.9 (70.1, 96.2) | 79.4 (66.8, 94.8) |
| **Height (cm)** | 168.2 (161.2, 176.1) | 164.9 (159.5, 171.7) | 168.5 (161.9, 176.0) | 170.6 (163.1, 177.8) | 170.5 (162.6, 178.1) | 166.5 (159.1, 174.9) |
| **Wasit (cm)** | 95.6 (85.5, 106.2) | 87.6 (79.1, 97.1) | 92.7 (83.5, 103.0) | 96.6 (87.2, 107.4) | 99.5 (89.2, 109.6) | 101.8 (93.2, 112.2) |
| **Smoking status (n, %)** |  |  |  |  |  |  |
| Never | 6,294.0 (50.1%) | 1,620.0 (63.5%) | 1,295.0 (52.1%) | 1,219.0 (48.8%) | 1,082.0 (42.6%) | 1,078.0 (41.9%) |
| Former | 3,355.0 (25.3%) | 454.0 (19.3%) | 593.0 (24.8%) | 634.0 (23.3%) | 753.0 (27.6%) | 921.0 (33.8%) |
| Current | 2,691.0 (24.6%) | 411.0 (17.3%) | 572.0 (23.0%) | 628.0 (27.9%) | 623.0 (29.9%) | 457.0 (24.2%) |
| **Drinking status (n, %)** |  |  |  |  |  |  |
| Never | 4,017.0 (27.4%) | 747.0 (24.9%) | 613.0 (21.5%) | 672.0 (22.8%) | 817.0 (29.1%) | 1,168.0 (44.2%) |
| Light | 4,320.0 (35.8%) | 970.0 (38.6%) | 893.0 (37.4%) | 905.0 (36.7%) | 824.0 (34.4%) | 728.0 (30.6%) |
| Moderate | 3,782.0 (34.9%) | 730.0 (35.1%) | 901.0 (39.2%) | 853.0 (38.6%) | 757.0 (33.7%) | 541.0 (24.2%) |
| Heavy | 221.0 (1.8%) | 38.0 (1.4%) | 53.0 (1.9%) | 51.0 (1.8%) | 60.0 (2.7%) | 19.0 (1.1%) |
| **TC (mg/L)** | 199.00 (174.00, 228.00) | 191.00 (169.00, 216.00) | 197.00 (170.00, 225.00) | 202.00 (176.00, 232.00) | 204.00 (180.00, 231.00) | 203.00 (177.00, 235.00) |
| **TG (mg/L)** | 128.00 (77.00, 203.00) | 106.24 (63.44, 179.51) | 117.09 (73.00, 195.73) | 129.00 (80.00, 200.55) | 142.00 (87.00, 212.62) | 154.00 (99.00, 229.00) |
| **LDL**-C **(mg/L)** | 120.00 (97.00, 143.00) | 115.00 (92.19, 138.00) | 119.00 (94.95, 142.00) | 123.00 (99.00, 145.00) | 121.47 (100.00, 144.61) | 120.91 (99.00, 143.12) |
| **HDL**-C **(mg/L)** | 50.00 (41.00, 61.00) | 56.00 (47.00, 68.00) | 52.00 (42.00, 62.00) | 47.00 (40.00, 59.00) | 46.00 (39.00, 56.00) | 46.00 (38.00, 58.00) |
| **Diabetes (n, %)** |  |  |  |  |  |  |
| Yes | 1,204.0 (6.9%) | 154.0 (4.2%) | 138.0 (3.9%) | 171.0 (4.9%) | 275.0 (8.1%) | 466.0 (16.0%) |
| No | 10,968.0 (92.0%) | 2,314.0 (95.2%) | 2,302.0 (95.6%) | 2,283.0 (94.1%) | 2,139.0 (90.5%) | 1,930.0 (81.4%) |
| Borderline | 168.0 (1.1%) | 17.0 (0.6%) | 20.0 (0.5%) | 27.0 (1.0%) | 44.0 (1.4%) | 60.0 (2.6%) |
| **Hypertension (n, %)** |  |  |  |  |  |  |
| Yes | 3,919.0 (27.4%) | 374.0 (13.3%) | 550.0 (20.5%) | 693.0 (24.0%) | 902.0 (32.5%) | 1,400.0 (53.7%) |
| No | 8,421.0 (72.6%) | 2,111.0 (86.7%) | 1,910.0 (79.5%) | 1,788.0 (76.0%) | 1,556.0 (67.5%) | 1,056.0 (46.3%) |
| **CAD (n, %)** |  |  |  |  |  |  |
| Yes | 561.0 (3.6%) | 20.0 (1.0%) | 44.0 (1.5%) | 57.0 (1.6%) | 129.0 (4.3%) | 311.0 (12.2%) |
| No | 11,779.0 (96.4%) | 2,465.0 (99.0%) | 2,416.0 (98.5%) | 2,424.0 (98.4%) | 2,329.0 (95.7%) | 2,145.0 (87.8%) |
| **Stroke (n, %)** |  |  |  |  |  |  |
| Yes | 424.0 (2.5%) | 18.0 (0.6%) | 33.0 (1.1%) | 46.0 (1.2%) | 93.0 (3.1%) | 234.0 (8.1%) |
| No | 11,916.0 (97.5%) | 2,467.0 (99.4%) | 2,427.0 (98.9%) | 2,435.0 (98.8%) | 2,365.0 (96.9%) | 2,222.0 (91.9%) |
| **Cancer** |  |  |  |  |  |  |
| Yes | 1,071.0 (8.2%) | 81.0 (3.7%) | 124.0 (5.3%) | 170.0 (6.8%) | 248.0 (9.9%) | 448.0 (17.8%) |
| No | 11,269.0 (91.8%) | 2,404.0 (96.3%) | 2,336.0 (94.7%) | 2,311.0 (93.2%) | 2,210.0 (90.1%) | 2,008.0 (82.2%) |
| **All cause of mortality** | 3,538.0 (20.6%) | 172.0 (5.2%) | 266.0 (8.0%) | 477.0 (13.7%) | 869.0 (25.6%) | 1,754.0 (62.4%) |
| **Cardivascular mortality** | 1,144.0 (6.3%) | 34.0 (0.9%) | 83.0 (2.6%) | 127.0 (3.6%) | 277.0 (7.5%) | 623.0 (21.2%) |
| **Cerebrovascular mortality (n, %)** | 202 (1.0%) | 6 (0.2%) | 19 (0.5%) | 17 (0.4%) | 61 (1.6%) | 99 (3.0%) |
| **Following-up time** | 207.0 (188.0, 227.0) | 205.0 (193.0, 226.0) | 213.0 (194.0, 230.0) | 213.0 (193.0, 230.0) | 209.0 (185.0, 227.0) | 163.2 (78.0, 212.0) |
| **Cystatin C (mg/L)** | 0.75 (0.66, 0.85) | 0.59 (0.56, 0.62) | 0.68 (0.66, 0.70) | 0.76 (0.74, 0.78) | 0.85 (0.82, 0.89) | 1.06 (0.99, 1.23) |
| **Creatinine (umol/L)** | 70.72 (61.88, 88.40) | 61.88 (53.04, 70.72) | 70.72 (61.88, 79.56) | 70.72 (61.88, 88.40) | 79.56 (70.70, 88.40) | 88.40 (70.72, 106.08) |
| **Creatinine group** |  |  |  |  |  |  |
| Q1: <53.05 | 2,721.00 (18.52%) | 1,106.00 (36.69%) | 628.00 (21.76%) | 484.00 (15.36%) | 337.00 (10.75%) | 166.00 (5.84%) |
| Q2: 53.05-70.70 | 2,617.00 (23.23%) | 577.00 (25.32%) | 615.00 (26.42%) | 576.00 (24.64%) | 537.00 (22.15%) | 312.00 (15.18%) |
| Q3: 70.71-79.56 | 3,053.00 (27.24%) | 567.00 (28.15%) | 741.00 (31.89%) | 709.00 (29.29%) | 654.00 (26.18%) | 382.00 (17.54%) |
| Q4: 79.57-88.40 | 1,828.00 (15.90%) | 152.00 (6.85%) | 287.00 (12.37%) | 406.00 (17.67%) | 502.00 (22.88%) | 481.00 (20.45%) |
| Q5: >88.40 | 2,121.00 (15.11%) | 83.00 (2.99%) | 189.00 (7.57%) | 306.00 (13.04%) | 428.00 (18.04%) | 1,115.00 (40.98%) |
| **eGFR (**ml/min1.73m^2^) | 98.79 (84.08, 112.89) | 111.90 (100.51, 121.94) | 105.23 (92.86, 116.75) | 99.46 (87.70, 112.10) | 91.44 (80.09, 104.35) | 70.06 (53.84, 87.63) |
| **eGFR group** |  |  |  |  |  |  |
| Q1: >117.52 | 2,464.00 (18.73%) | 1,082.00 (35.57%) | 648.00 (23.80%) | 430.00 (17.55%) | 227.00 (10.03%) | 77.00 (3.32%) |
| Q2: 103.01-117.52 | 2,470.00 (24.48%) | 741.00 (35.23%) | 715.00 (32.11%) | 573.00 (25.92%) | 351.00 (17.59%) | 90.00 (6.71%) |
| Q3: 90.49-103.00 | 2,466.00 (21.36%) | 412.00 (17.74%) | 588.00 (23.49%) | 661.00 (25.62%) | 576.00 (24.74%) | 229.00 (11.77%) |
| Q4: 75.00-90.48 | 2,472.00 (20.47%) | 205.00 (9.61%) | 420.00 (17.66%) | 587.00 (23.12%) | 781.00 (30.57%) | 479.00 (20.66%) |
| Q5: <75.00 | 2,468.00 (14.96%) | 45.00 (1.84%) | 89.00 (2.94%) | 230.00 (7.79%) | 523.00 (17.06%) | 1,581.00 (57.54%) |

* Numbers of participants were unweighted, and all percentage estimates are weighted. Other data are weighted estimates, and expressed as median (percentile 25, percentile 75).

**Wilcoxon rank-sum test for complex survey samples; chi-squared test with Rao & Scott's second-order correction

BMI: body mass index, TC: total cholesterol, TG: triglyceride, LDL-C: low-density lipoprotein-cholesterol, HDL-C: high-density lipoprotein-cholesterol, CAD: coronary artery disease, eGFR: estimate Glomerular Filtration Rate. eGFR was calculated using the CKD-EPI creatitine equation.

**Table 2 Risk of adverse outcomes according to Cystatin C levels**

|  | **Crude Model** | | | **Model 1** | | | **Model 2** | | | **Model 3** | | |
| --- | --- | --- | --- | --- | --- | --- | --- | --- | --- | --- | --- | --- |
|  | **HR** | **95% CI** | ***P* value** | **HR** | **95% CI** | ***P* value** | **HR** | **95% CI** | ***P* value** | **HR** | **95% CI** | ***P* value** |
| **All-cause of mortality** |  |  |  |  |  |  |  |  |  |  |  |  |
| **Cystatin per 0.1mg/L** | 1.08 | 1.07, 1.09 | <0.001 | 1.06 | 1.06, 1.07 | <0.001 | 1.06 | 1.05, 1.07 | <0.001 | 1.06 | 1.05, 1.06 | <0.001 |
| **Cystatin C group** |  |  |  |  |  |  |  |  |  |  |  |  |
| Q1: <0.640 mg/L | Ref. |  |  | Ref. |  |  | Ref. |  |  | Ref. |  |  |
| Q2: 0.640–0.717 mg/L | 1.53 | 1.21, 1.93 | <0.001 | 1.08 | 0.86, 1.37 | 0.510 | 1.04 | 0.82, 1.32 | 0.743 | 1.05 | 0.82, 1.34 | 0.685 |
| Q3: 0.718–0.799 mg/L | 2.68 | 2.14, 3.35 | <0.001 | 1.33 | 1.06, 1.66 | 0.014 | 1.25 | 1.01, 1.56 | 0.045 | 1.29 | 1.03, 1.60 | 0.024 |
| Q4: 0.800–0.932 mg/L | 5.30 | 4.23, 6.63 | <0.001 | 1.62 | 1.30, 2.00 | <0.001 | 1.46 | 1.17, 1.83 | <0.001 | 1.46 | 1.17, 1.83 | <0.001 |
| Q5: >0.932 mg/L | 18.30 | 15.20, 22.2 | <0.001 | 2.76 | 2.25, 3.39 | <0.001 | 2.40 | 1.93, 2.98 | <0.001 | 2.32 | 1.86, 2.89 | <0.001 |
| ***P* for trend** |  |  | <0.001 |  |  | <0.001 |  |  | <0.001 |  |  | <0.001 |
| **Cardiovascular mortality** |  |  |  |  |  |  |  |  |  |  |  |  |
| **Cystatin per 0.1mg/L** | 1.08 | 1.07, 1.09 | <0.001 | 1.07 | 1.06, 1.08 | <0.001 | 1.07 | 1.06, 1.08 | <0.001 | 1.06 | 1.05, 1.07 | <0.001 |
| **Cystatin C group** |  |  |  |  |  |  |  |  |  |  |  |  |
| Q1: <0.640 mg/L | Ref. |  |  | Ref. |  |  | Ref. |  |  | Ref. |  |  |
| Q2: 0.640–0.717 mg/L | 2.76 | 1.72, 4.42 | <0.001 | 1.83 | 1.14, 2.96 | 0.013 | 1.70 | 1.05, 2.75 | 0.030 | 1.78 | 1.11, 2.86 | 0.018 |
| Q3: 0.718–0.799 mg/L | 3.97 | 2.38, 6.63 | <0.001 | 1.71 | 1.02, 2.87 | 0.041 | 1.55 | 0.93, 2.60 | 0.093 | 1.64 | 0.99, 2.72 | 0.053 |
| Q4: 0.800–0.932 mg/L | 8.69 | 5.41, 14.0 | <0.001 | 2.13 | 1.33, 3.41 | 0.002 | 1.79 | 1.12, 2.86 | 0.015 | 1.85 | 1.17, 2.92 | 0.009 |
| Q5: >0.932 mg/L | 35.00 | 22.1, 55.2 | <0.001 | 3.89 | 2.45, 6.16 | <0.001 | 3.00 | 1.91, 4.72 | <0.001 | 2.97 | 1.89, 4.68 | <0.001 |
| ***P* for trend** |  |  | <0.001 |  |  | <0.001 |  |  | <0.001 |  |  | <0.001 |
| **Cerebrovasculard mortality** |  |  |  |  |  |  |  |  |  |  |  |  |
| **Cystatin per 0.1mg/L** | 1.08 | 1.07, 1.09 | <0.001 | 1.06 | 1.04, 1.07 | <0.001 | 1.05 | 1.03, 1.07 | <0.001 | 1.04 | 1.02, 1.06 | <0.001 |
| **Cystatin C group** |  |  |  |  |  |  |  |  |  |  |  |  |
| Q1: <0.640 mg/L | Ref. |  |  | Ref. |  |  | Ref. |  |  | Ref. |  |  |
| Q2: 0.640–0.717 mg/L | 2.89 | 0.85, 9.85 | 0.09 | 1.86 | 0.54, 6.35 | 0.322 | 2.05 | 0.59, 7.15 | 0.259 | 2.09 | 0.60, 7.28 | 0.245 |
| Q3: 0.718–0.799 mg/L | 2.15 | 0.55, 8.37 | 0.27 | 0.86 | 0.22, 3.26 | 0.819 | 1.03 | 0.27, 3.94 | 0.961 | 1.06 | 0.28, 4.00 | 0.937 |
| Q4: 0.800–0.932 mg/L | 9.05 | 2.86, 28.6 | <0.001 | 1.94 | 0.63, 5.94 | 0.248 | 2.31 | 0.77, 6.95 | 0.135 | 2.3 | 0.76, 6.95 | 0.142 |
| Q5: >0.932 mg/L | 24.10 | 7.05, 82.0 | <0.001 | 2.15 | 0.64, 7.28 | 0.219 | 2.52 | 0.75, 8.46 | 0.134 | 2.41 | 0.71, 8.24 | 0.16 |
| ***P* for trend** |  |  | <0.001 |  |  | 0.217 |  |  | 0.153 |  |  | 0.214 |

Model 1: Adjusted for age and sex. Model 2: Model 1 plus BMI, race, smoking and drinking status. Model 3: Model 2 plus history of hypertension, diabetes, coronary artery disease, stroke and cancer.

**Table 3 Associations of cystatin C groups with all-cause, cardiovascular, and cerebrovascular mortality**

|  | **Crude Model** | | | **Model 1** | | | **Model 2** | | | **Model 3** | | |
| --- | --- | --- | --- | --- | --- | --- | --- | --- | --- | --- | --- | --- |
|  | **HR** | **95% CI** | **P value** | **HR** | **95% CI** | **P value** | **HR** | **95% CI** | **P value** | **HR** | **95% CI** | **P value** |
| **All-cause of mortality** |  |  |  |  |  |  |  |  |  |  |  |  |
| **Cystatin group <0.718 mg/L** | Ref. |  |  | Ref. |  |  | Ref. |  |  | Ref. |  |  |
| **0.718-0.932 mg/L** | 3.03 | 2.60, 3.54 | <0.001 | 1.41 | 1.21, 1.64 | <0.001 | 1.33 | 1.15, 1.54 | <0.001 | 1.34 | 1.15, 1.55 | <0.001 |
| **>0.932 mg/L** | 14.30 | 12.40, 16.40 | <0.001 | 2.58 | 2.24, 2.99 | <0.001 | 2.30 | 2.00, 2.65 | <0.001 | 2.22 | 1.91, 2.58 | <0.001 |
| **Cardivascular mortality** |  |  |  |  |  |  |  |  |  |  |  |  |
| **Cystatin group <0.718 mg/L** | Ref. |  |  | Ref. |  |  | Ref. |  |  | Ref. |  |  |
| **0.718-0.932 mg/L** | 3.16 | 2.31, 4.33 | <0.001 | 1.27 | 0.93, 1.73 | 0.138 | 1.16 | 0.85, 1.60 | 0.347 | 1.16 | 0.84, 1.60 | 0.353 |
| **>0.932 mg/L** | 17.90 | 13.40, 24.00 | <0.001 | 2.48 | 1.82, 3.37 | <0.001 | 2.12 | 1.56, 2.87 | <0.001 | 1.94 | 1.40, 2.67 | <0.001 |
| **Cerebrovascular mortality** |  |  |  |  |  |  |  |  |  |  |  |  |
| **Cystatin group <0.718mg/L** | Ref. |  |  | Ref. |  |  | Ref. |  |  | Ref. |  |  |
| **0.718-0.932 mg/L** | 2.65 | 1.50, 4.69 | <0.001 | 0.95 | 0.54, 1.65 | 0.847 | 1.04 | 0.60, 1.81 | 0.889 | 1.04 | 0.59, 1.81 | 0.904 |
| **>0.932 mg/L** | 11.90 | 6.66, 21.2 | <0.001 | 1.3 | 0.72, 2.35 | 0.390 | 1.44 | 0.79, 2.63 | 0.236 | 1.31 | 0.70, 2.45 | 0.398 |

Model 1: Adjusted for age and sex. Model 2: Model 1 plus BMI, race, smoking and drinking status. Model 3: Model 2 plus history of hypertension, diabetes, coronary artery disease, stroke and cancer.

**Fig 1. Flowchart depicting the study design**

**Fig 2. Number of adverse events within each group according to quintiles of cystatin C, creatinine, and eGFR**

**Fig 3. Kaplan–Meier curve.** Weighted Kaplan–Meier curves assessed the differences across cystatin C quintiles.

**Fig 4. Restricted cubic spline curves showing the associations of serum cystatin C levels with all-cause (A), cardiovascular (B), and cerebrovascular (C) mortality.** Splines were fitted with five knots placed at the 5th, 27.5th, 50th, 72.5th, and 95th percentiles of cystatin C distribution. Adjusted hazard ratios (95% CIs) were derived from Cox proportional hazards models, adjusted for age, sex, body mass index, race, smoking status, drinking status, and histories of hypertension, diabetes, coronary artery disease, stroke, and cancer. Histograms represent the probability density of cystatin C levels in the study population.
